# Lithium protects hippocampal progenitors, cognitive performance and hypothalamus–pituitary function after irradiation to the juvenile rat brain

**DOI:** 10.18632/oncotarget.16292

**Published:** 2017-03-16

**Authors:** Kai Zhou, Cuicui Xie, Malin Wickström, Amalia M. Dolga, Yaodong Zhang, Tao Li, Yiran Xu, Carsten Culmsee, Per Kogner, Changlian Zhu, Klas Blomgren

**Affiliations:** ^1^ Centre for Brain Repair and Rehabilitation, Institute of Neuroscience and Physiology, University of Gothenburg, Gothenburg, Sweden; ^2^ Karolinska Institutet, Department of Women's and Children's Health, Stockholm, Sweden; ^3^ Henan Key Laboratory of Child Brain Injury, The Third Affiliated Hospital of Zhengzhou University, Zhengzhou, China; ^4^ Institute of Pharmacology and Clinical Pharmacy, University of Marburg, Marburg, Germany; ^5^ Department of Molecular Pharmacology, University of Groningen, Groningen Research Institute of Pharmacy, Groningen, The Netherlands; ^6^ Department of Paediatrics, Zhengzhou Children's Hospital, Zhengzhou, China; ^7^ Department of Paediatric Oncology, Karolinska University Hospital, Stockholm, Sweden

**Keywords:** brain tumour, cell death, late effects, neuroinflammation, radiotherapy

## Abstract

Cranial radiotherapy in children typically causes delayed and progressive cognitive dysfunction and there is no effective preventive strategy for radiation-induced cognitive impairments. Here we show that lithium treatment reduced irradiation-induced progenitor cell death in the subgranular zone of the hippocampus, and subsequently ameliorated irradiation-reduced neurogenesis and astrogenesis in the juvenile rat brain. Irradiation-induced memory impairment, motor hyperactivity and anxiety-like behaviour were normalized by lithium treatment. Late-onset irradiation-induced hypopituitarism was prevented by lithium treatment. Additionally, lithium appeared relatively toxic to multiple cultured tumour cell lines, and did not improve viability of radiated DAOY cells in vitro. In summary, our findings demonstrate that lithium can be safely administered to prevent both short- and long-term injury to the juvenile brain caused by ionizing radiation.

## INTRODUCTION

Brain tumours are the most frequent paediatric solid tumours and they represent the leading cause of mortality and morbidity by cancer in this age group [1]. Current treatment protocols for malignant paediatric brain tumours, the most common of which is medulloblastoma, typically include surgery, irradiation, and chemotherapy, a lifesaving combination that unfortunately contributes to long-term physical, endocrine, and neuropsychological impairments in survivors [2]. Radiotherapy often causes delayed and progressive cognitive impairments, such as deficits in memory, attention, and executive function [3, 4] with tremendous impact on the patients’ quality of life. Cognitive function is one of the most important measurements of brain tumour therapy outcomes in clinical trials, second only to survival [5]. Clinical studies suggest that radiation-induced damage to the hippocampus plays a significant role in cognitive deficits [6–8]. Animal studies have shown that irradiation reduces hippocampal neurogenesis, and the mechanisms involved include apoptosis, neuroinflammation, and oxidative stress [9–11]. In addition, systemic effects of cranial irradiation, especially radiation-induced endocrinopathies, influence mental and physical development [12].

Efforts have been made to prevent or ameliorate the side effects induced by cerebral irradiation, for example by targeting different signalling pathways [13, 14]. Clinical trials using memantine, a competitive N-methyl-D-aspartate (NMDA) receptor antagonist, exerted radioprotective effects in adult patients [15, 16], but as of yet there are no successful long-term treatments or effective preventive strategies safely used for radiation-induced cognitive impairments in children or adolescents [17, 18]. The only strategy that has been tested in a clinical setting is physical exercise. Exercise is known to partly restore hippocampal neurogenesis and behaviour even when initiated long after irradiation in mice [11], and three very recent studies indicate that this may be effective also in children and young adults [19–21]. Regarding pharmacological strategies, lithium, when compared with memantine [22] or CNS stimulants, appears to exert multiple effects that reduce the negative effects of ionizing radiation. Several studies have indicated that lithium has robust neuroprotective effects in cranial irradiation paradigms [9, 23–25]. The protective properties include inhibition of stem or progenitor cell death and neuroinflammation [26], rescue of synaptic plasticity [27], and stimulation of stem and progenitor cell proliferation and neurogenesis [9, 28]. Importantly, lithium does not appear to protect cancer cells [23] and may in some cases act as a radiosensitizer [29]. Before moving to clinical trials of lithium for prevention of radiation-induced cognitive impairments, it is important to assess its long-term effects on irradiation-induced brain injury, as well as systemic effects, mechanisms of action, and potential toxicity. Neurogenesis in the dentate gyrus of the hippocampus is important in long term memory formation, and it has been shown that ionizing radiation effectively impairs neurogenesis [9, 11]. We have earlier found that lithium treatment effectively reduces ischemic as well as irradiation injury [9, 26, 28, 30, 31]. The purpose of this study was to test our hypothesis that lithium treatment could ameliorate irradiation-induced so called late effects, such as cognitive decline and endocrine dysfunction, by preventing cell death and promoting regeneration.

## RESULTS

### Tumour cells are sensitive to lithium toxicity

**Figure 1:**
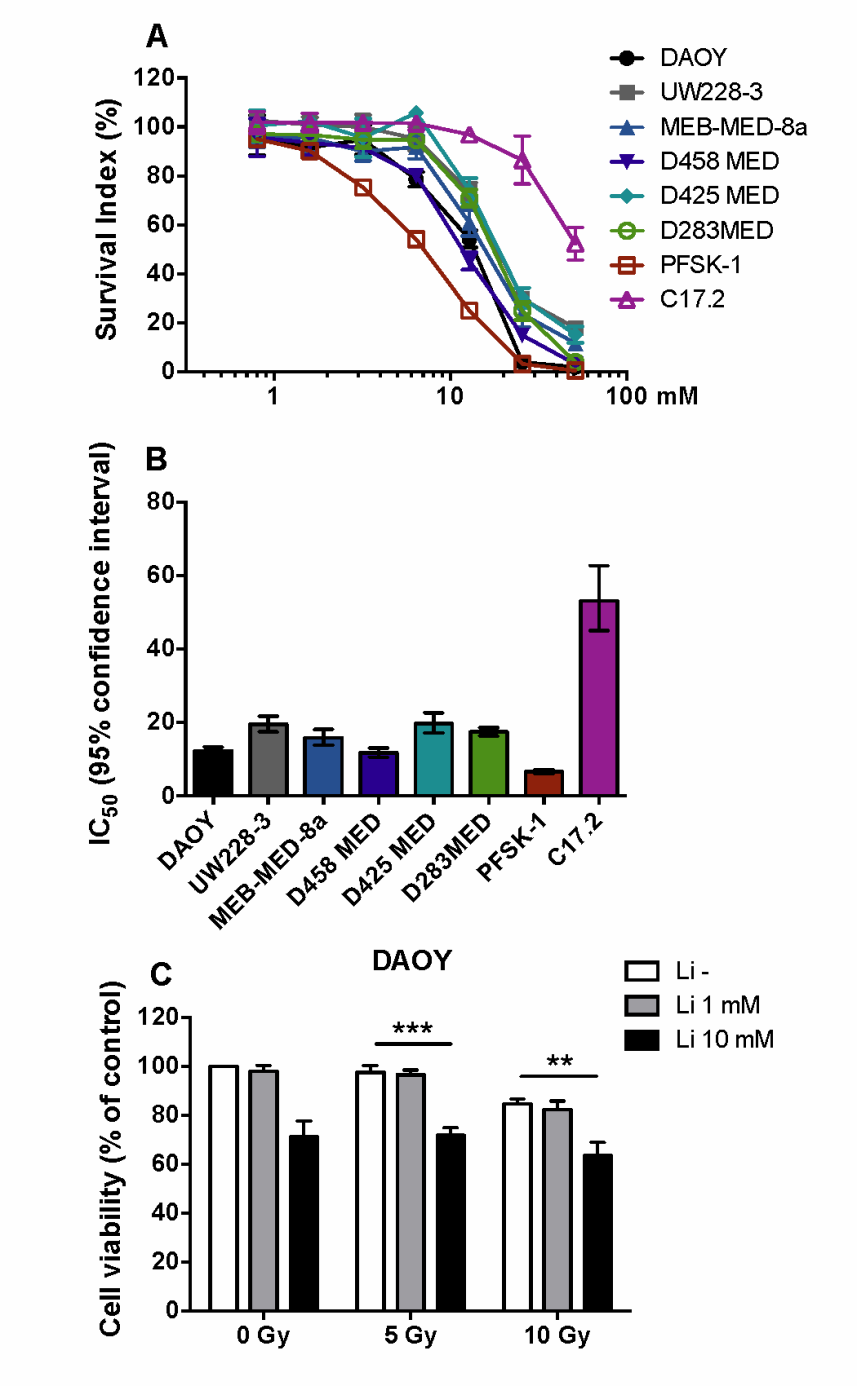
Dose-dependent lithium toxicity *in vitro*. **A**. Dose-response curve for lithium toxicity showing that the neural multipotent stem/progenitor cell line C17.2 is far more resistant to lithium toxicity than 7 different paediatric malignant brain tumour cell lines (72 h). **B**. A bar graph showing the IC_50_ and 95% confidence interval values of lithium for the 8 different cell lines. The IC_50_ of the neural multipotent progenitor cell line is 2.7-8.1 times higher than that of the tumour cell lines. **C**. The effect of lithium and irradiation on cell survival of DAOY cells, demonstrating that lithium has no protective effect against irradiation. ** *p* < 0.01, *** *p* < 0.001.

To examine the sensitivity difference of human tumour cells to lithium treatment, 7 brain tumour cell lines were treated with lithium at different concentrations, and as a comparison a neural multipotent progenitor cell line was included in the analysis. The survival of tumour cells decreased dramatically when the lithium concentration was more than 12.8 mM, but no significant change for the immortalised neural progenitor cells was observed (Figure 1A). The IC_50_ values for the tumour cell lines varied between 6.5 to 20 µM (Figure 1B). We further examined the combined effects of lithium and irradiation on DAOY cells, a medulloblastoma cell line which is resistant to irradiation. In this tumour cell line, cell viability decreased significantly in the presence of 10 mM lithium (*p* < 0.01), and no protective effect of lithium on the tumour cells was observed (Figure 1C).

### Effects of lithium treatment on cell death in the DG after irradiation

**Figure 2:**
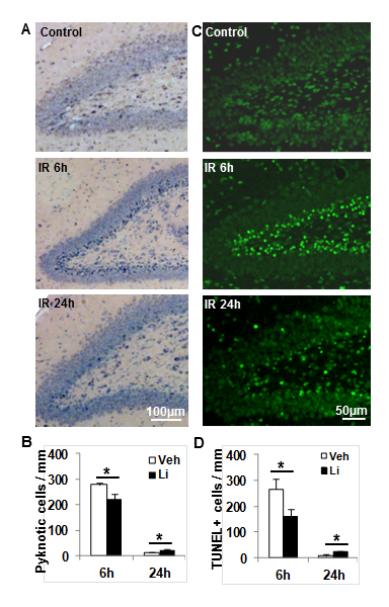
Cell death in the DG at 6 h and 24 h after irradiation **A**. Representative photomicrograph of H&E staining in the DG at 6 h and 24 h after irradiation. **B**. The bar graph shows the quantification of pyknotic cells in the SGZ at 6 h and 24 h after irradiation in both vehicle-treated and lithium-treated rats. **C**. Representative TUNNEL staining in the DG at 6 h and 24 h after irradiation. **D**. Quantification of TUNEL-labelled cells in the vehicle-treated and lithium-treated rats after irradiation. *n* = 6/group for the 6 h vehicle and lithium treatment, *n* = 6 for the vehicle group and *n* = 7 for the lithium treatment group at 24 h. * *p* < 0.05.

Pyknotic cells were detected in the SGZ 6 h after irradiation using H&E staining (Figure 2A) and the number of the pyknotic cells was 19.8% lower after lithium treatment (*p* < 0.05) (Figure 2B). As expected, because of rapid clearance the overall number of pyknotic cells was lower 24 h after irradiation compared to the earlier (6 h) time point [32]. At this later time point, however, the number of pyknotic cells was higher in the lithium-treated group compared to the vehicle-treated group (*p* < 0.05) (Figure 2B), presumably indicating not only inhibited, but also some degree of delayed, cell death in the lithium-treated group. Similar tendencies were observed after TUNEL labelling (Figure 2C) and quantification of TUNEL-labelled cells (Figure 2D).

### Effects of lithium treatment on proliferation and differentiation in the DG

**Figure 3:**
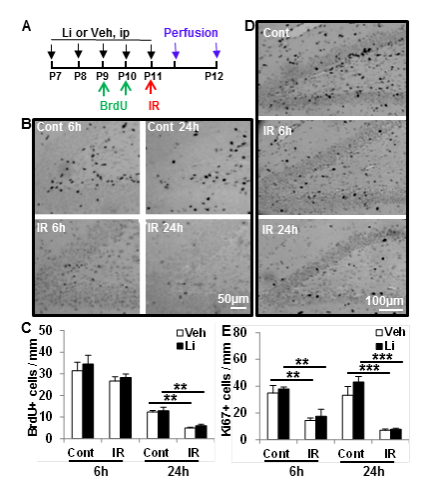
Short- term effect of lithium on cell proliferation **A**. The study design. **B**. Representative photomicrographs of BrdU staining in the DG at 6 h and 24 h after irradiation. **C**. Quantification of BrdU-positive cells in the SGZ of the DG in the control (Cont) and ipsilateral hemisphere after irradiation (IR) in vehicle-treated and lithium-treated animals. **D**. Representative microphotographs of Ki67 staining in the SGZ at 6 h and 24 h after irradiation. **E**. Quantification of Ki67-positive cells in the SGZ of the DG in the control and irradiated rats with vehicle treatment and lithium treatment (*n* = 6/group for 6 h, *n* = 6 for the vehicle group and *n* = 7 for the lithium group at 24 h). ***p < 0.001.

The short-term effects of lithium treatment on proliferation and cell survival were investigated by counting BrdU- and Ki67-labelled cells. The number of BrdU-labelled cells was slightly decreased at 6 h and significantly decreased at 24 h after irradiation compared with non-irradiated controls, but no difference was seen between vehicle and lithium treatment groups (Figure 3A-3C). Ki67-labelled cells decreased 6 h after irradiation compared to the non-irradiated controls, and this decrease was more pronounced 24 h after irradiation, but lithium had no effect on this irradiation-induced reduction of proliferating cells (Figure 3D-3E).

**Figure 4:**
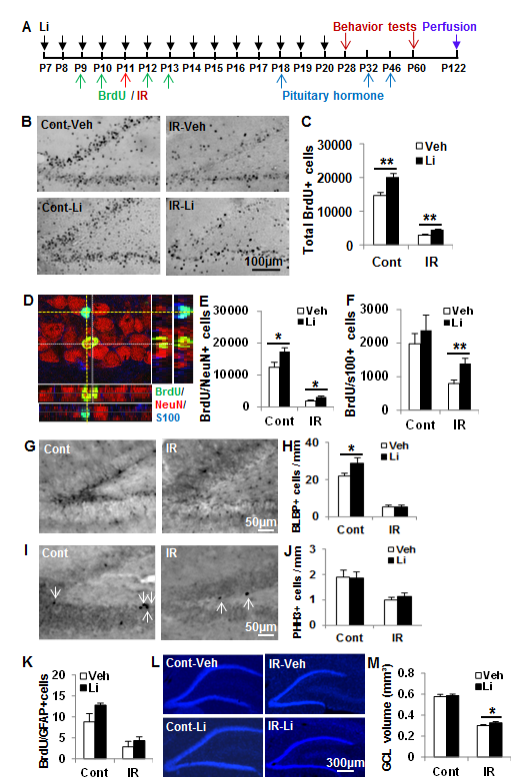
Effect of lithium on proliferated cell survival and differentiation after irradiation **A**. The study design. **B**. Representative BrdU staining from the dorsal hippocampal level at 16 weeks after irradiation. **C**. Quantification of BrdU-labelled cells in the GZ of the DG at 16 weeks after irradiation. **D**. Representative BrdU-NeuN-S100 staining. **E**. The quantification of newborn neurons in the DG. **F**. The quantification of newborn astrocytes in the DG. **G**. Representative microphotographs of BLBP staining in the DG at 16 weeks after irradiation. **H**. The bar graph shows quantification of BLBP-positive cells in the SGZ of the DG. **I**. Representative microphotographs of PHH3 staining in the SGZ at 16 weeks after irradiation. **J**. The bar graph shows quantification of PHH3-positive cells in the SGZ of the DG. **K**. The graph shows the absolute numbers of BrdU-GFAP double-labelled cells in the SGZ of the DG at 16 weeks after irradiation. **L**. Representative DAPI staining of the DG of the hippocampus. **M**. Quantification of the granular cell layer (GCL) volume of the DG showing a reduction of about 44% in lithium-treated rats and 48% in vehicle-treated rats (*n* = 14 for control vehicle, *n* = 16 for control lithium, *n* = 16 for irradiation vehicle, *n* = 18 for irradiation lithium). * *p* < 0.05, ** *p* < 0.01.

The long-term effects of lithium treatment on survival of BrdU-labelled cells and differentiation were investigated by counting and phenotyping BrdU-labelled cells 16 weeks after irradiation (Figure 4A). The total number of surviving BrdU-labelled cells per DG after 16 weeks in the non-irradiated lithium-treated group was 35.6% higher than in the vehicle-treated group (*p* < 0.01) (Figure 4B-4C). The number of surviving BrdU-labelled cells in the irradiated animals was decreased dramatically compared with the non-irradiated controls, but the total number of surviving BrdU-labelled cells after irradiation was 50.1% higher in the lithium-treated group compared to the vehicle-treated group (Figure 4B-4C). The phenotyping of newly generated cells surviving at least 16 weeks showed that neurogenesis (BrdU+/NeuN+ cells) was higher in lithium-treated animals, both in the non-irradiated (39.5%) and irradiated rats (64.5%) (Figure 4D-4E). The number of newly generated astrocytes (BrdU+/S100+ cells) was not significantly different between lithium-treated and vehicle-treated non-irradiated rats, but it was higher (75.2%) in the lithium-treated group compared to the vehicle-treated group after irradiation (Figure 4F). Increased numbers of neural stem cells (BLBP+ cells) after lithium treatment was observed in non-irradiated, but not in irradiated, brains 16 weeks later (Figure 4G-4H). The ratio of neurogenesis to astrogenesis was not different between the lithium-treated and vehicle-treated groups, indicating that lithium treatment resulted in increased overall survival of stem or progenitor cells (NSPCs) and did not specifically promote one cell fate over the other. The number of proliferating (PHH3+) cells at this late time point was reduced by irradiation, but not altered by lithium (Figure 4I-4J). To further check if lithium had an effect on the neural stem cell pool, we performed BrdU-GFAP double labelling, and counted double-positive cells in the SGZ. Although there was a tendency towards an increase in the non-irradiated brains, as for BLBP, there was no significant difference between vehicle- and lithium-treated mice, neither in controls, nor in irradiated brains (Figure 4K). Hence, irradiation induced persistent inhibition of NSPC proliferation after irradiation, and lithium had no apparent effect on this in the irradiated rats, despite reduced cell death and increased neurogenesis and astrogenesis. The volumes of the GCL in the DG was measured based on DAPI staining (Figure 4L), and the volume was decreased by 48.1% 16 weeks after irradiation in the vehicle-treated group and by 44.3% in the lithium-treated group. The total GCL volume was not significantly different in the non-irradiated control groups, but was 9.4% higher in the lithium-treated irradiated group compared to the vehicle-treated irradiated group (*p* < 0.05) (Figure 4M).

### Effects of lithium on microglia activation and inflammation in the hippocampus after irradiation

**Figure 5:**
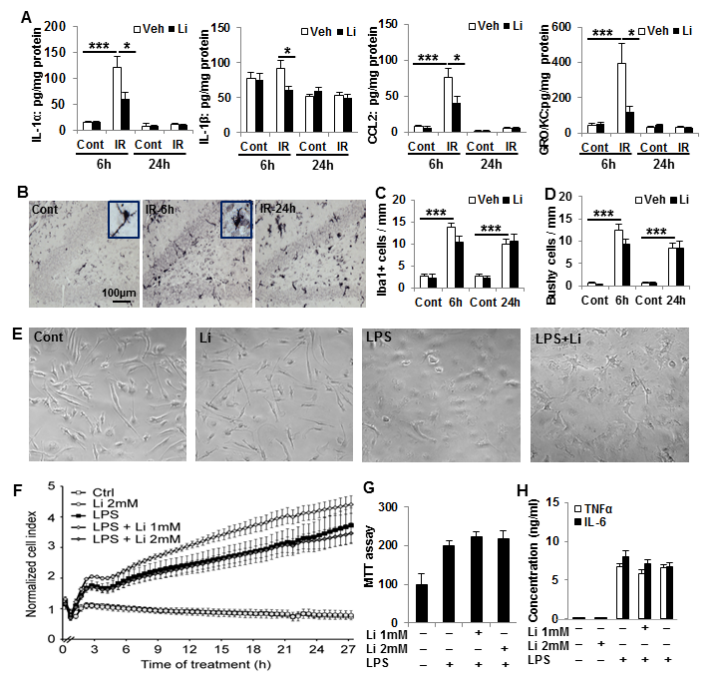
Lithium prevents irradiation-induced early inflammation in the hippocampus, but not effect on microglia activation *in vivo* and *in vitro* **A**. IL-1α, MCP-1, and GRO/KC increased dramatically at 6 h after irradiation compared to the non-irradiated controls. Lithium treatment prevented increases in IL-1α, MCP-1, and GRO/KC (D). The level of IL-1β was lower in the irradiated pups with lithium treatment at 6 h, but not at 24 h (*n* = 5 for 6 h control, *n* = 7 for 6 h irradiation, *n* = 4 for 24 h control, and n = 5 for 24 h IR). **B**. Representative Iba1 immunostaining of normal control and 6h, 24h after irradiation in the DG of hippocampus. **C**. Bar graph shows Iba1 positive cells increased significantly after irradiation compared to no-irradiated controls. **D**. Quantification of bushy cells based on the Iba1 staining and morphology. Most of the Iba1 positive cells were bushy morphology after irradiation. **E**. Representative pictures of microglia morphology *in vitro* under the four different conditions. **F**. xCELLigence analysis of microglia cells treated with LPS and/or lithium. **G**. Viability (MTT) analysis of microglia treated with LPS and lithium. The number and/or size of microglia increased after LPS treatment, and lithium had no effect on this. **H**. A bar graph of TNFα and IL-6 concentrations in the microglia culture medium. LPS stimulates TNFα and IL-6 secretion, and lithium had no apparent effect on this. *p < 0.05, ***p < 0.001.

Cytokines (IL-1α, IL-1β) and chemokines (CCL2, GRO/KC) increased significantly in hippocampal tissue 6 h after irradiation, except IL-1β, and lithium treatment prevented the irradiation-induced hippocampal inflammatory response (Figure 5A). The irradiation-induced inflammatory reaction was transient and had returned to non-irradiated control levels by 24 h after irradiation. Unlike the 6 h time point, there were no differences between vehicle-treated and lithium-treated groups at 24 h time point (Figure 5A). The number of microglia increased dramatically in the GCL after irradiation, and microglia were activated, as judged by their morphology, but lithium had no apparent effect on either of these two parameters (Figure 5B-5D). To further analyse the effects of lithium on microglia activation and inflammation, primary microglia were cultured, treated with lithium and stimulated with LPS. Cell numbers and morphological alterations were monitored by real-time impedance using an xCELLigence system. LPS induced a steady impedance increase, mainly attributed to changes in microglial shape, first detected 3 h following LPS exposure. This increase in cell index persisted during the following 24 h (Figure 5E-5F). Lithium treatment alone did not alter microglial shape under resting conditions. Moreover, pre-treatment with lithium for 24 h followed by LPS challenge did not change the LPS-induced cell index increase (Figure 5F). Furthermore, MTT cell proliferation analysis showed that lithium pre- and co-treatment did not affect LPS-induced microglia proliferation (Figure 5G). Neither IL-6 nor TNF-α (Figure 5H) were detected in the medium of non-stimulated microglia, but both of them increased in response to LPS, and lithium treatment had no effect on these cytokine levels. In summary, lithium did not appear to have any direct, anti-inflammatory effect of microglia. This indicates that the reduced inflammatory response *in vivo* likely was secondary to the reduced injury.

**Figure 6:**
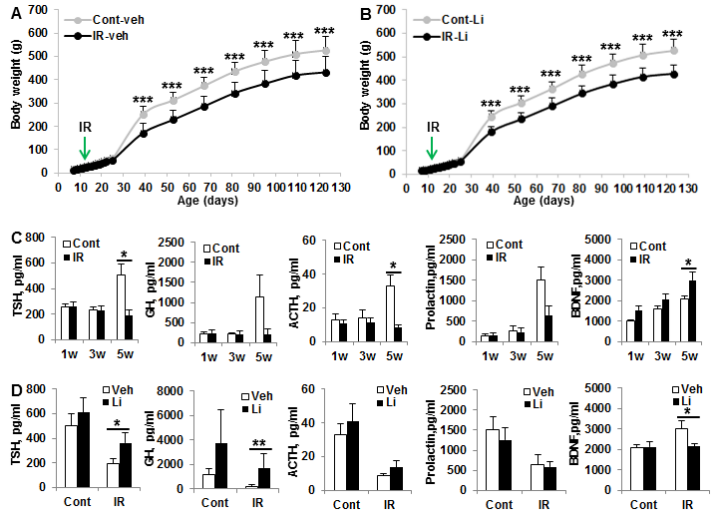
Effect of lithium treatment on pituitary hormones and body weight gain **A**. Body weight in the vehicle-treated control and irradiated rats showed body weight gain was slow at 4 weeks, and this difference was still significant at 16 weeks after irradiation. **B**. Similar trend in body weight gain in lithium-treated rats as for the vehicle-treated rats (*n* = 14 for control vehicle, *n* = 16 for control lithium, *n* = 16 for irradiation vehicle, and *n* = 18 for irradiation lithium). **C**. The pituitary hormones TSH, ACTH, prolactin and BDNF in serum increased with age and were significantly higher at about 6 weeks after birth, and irradiation prevented the developmentally associated increases in pituitary hormones. **D**. Lithium treatment prevented irradiation-induced pituitary TSH reduction and BDNF increase at 5 weeks after irradiation (*n* = 6/group). *p < 0.05, ** *p* < 0.01, *** *p* < 0.001

Effects of lithium on body weight gain and pituitary-related hormones after irradiation Body weight gain was delayed in irradiated rats, detectable from about 4 weeks after irradiation (Figure 6A, 6B). This effect was persistent, but lithium treatment had no influence on body weight gain, neither in normal control rats, nor in irradiated animals. Serum creatinine levels were increased during the rapid growth period, but no difference was seen between the vehicle-treated and lithium-treated animals at any time point (data not shown). Pituitary-related hormones, including thyroid-stimulating hormone (TSH), ACTH, prolactin, growth hormone (GH) and brain-derived neurotrophic factor (BDNF) were assayed in serum at different time points after irradiation. All these hormones increased 2- to 10-fold when the body growth rate increased, but the normal, growth-related increase in pituitary hormones was reduced or even abolished in irradiated rats (Figure 6C). Lithium treatment restored TSH and GH levels, and reduced the injury-induced BDNF increase [33], indicating that the injury to the hypothalamus–pituitary axis was ameliorated, thereby reducing the risk of developing hypothyroidism and GH deficiency (Figure 6D).

### Effect of lithium on motor activity and recognition memory

**Figure 7:**
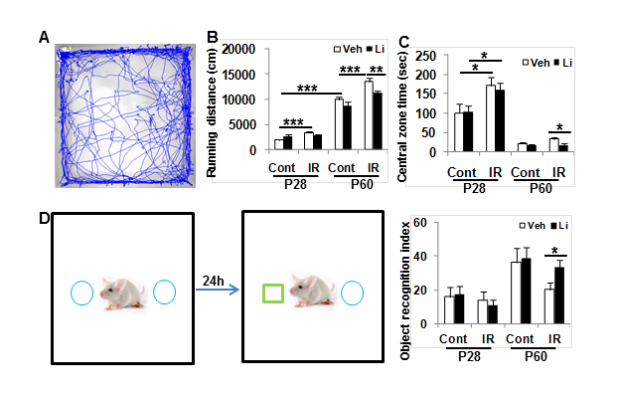
Behavioural tests **A**. Open field tests show the accumulated distances moved over 20 min at P28 and P60. **B.** The irradiated rats were more active than the controls. Lithium treatment normalized the motor activity. **C**. Accumulated time spent in the open central zone in a 20-min open field test. The irradiated rats spent more time in the open central zone at P28 compare to non-irradiated controls. Lithium treatment reduced time spent in the open central zone in the irradiated rats at P60. **D**. The novel object recognition test showed that irradiation prevented recognition development and that lithium treatment normalized the irradiation-induced recognition deficit. *n* = 14 for control vehicle, *n* = 16 for control lithium, *n* = 16 for irradiation vehicle, and *n* = 18 for irradiation lithium. * *p* < 0.05, ** *p* < 0.01, *** p < 0.001.

Motor activity increased with age, as indicated by running distance (*p* < 0.0001) (Figure 7A-7B). The accumulated running distance in P28 rats was 74.5% greater for the irradiated rats compared to the non-irradiated controls (*p* < 0.001) and 35.6% greater for P60 rats (*p* < 0.001). Lithium treatment reduced irradiation-induced hyperactivity in P60 rats (*p* < 0.01) but not in P28 rats. The time spent in the open central zone was 71.4% greater in the irradiated rats compared to the non-irradiated controls (*p* < 0.05). Lithium treatment normalised the time spent in the open central zone in irradiated P60 rats (*p* < 0.05) (Figure 7C). Memory function, as judged by novel object recognition, increased with age between P28 and P60 in normal control rats (*p* < 0.05). Irradiation had no obvious effect on recognition memory in P28 rats, but there was a substantial decrease in memory function in irradiated P60 rats, and this deficit was normalized by lithium treatment (*p* < 0.05) (Figure 7D).

## DISCUSSION

In this study we found that lithium was toxic to all the 7 different human paediatric brain tumour cell lines tested, but the IC_50_ lithium concentrations *in vitro* were in the order of one magnitude higher than the therapeutical serum concentrations used. The intra- and extracellular lithium concentrations required to obtain the observed effects in the brain are not known, but we recently showed that serum concentrations of 1.2 mM in mice yielded brain tissue concentrations in the range of 0.82 - 0.05 mM, where the highest concentrations were observed in the neurogenic regions [34]. Lithium did not appear to protect medulloblastoma cells from radiation-induced cell death *in vitro*. This is line with the findings using mouse hippocampal neuronal HT-22 cells and mouse glioma cells [23]. Lithium has even been shown to abrogate TP53-associated radiation resistance in medulloblastomas, thereby enhancing the anti-tumour effects of radiotherapy [29]. However, we have previously demonstrated the ability of lithium to reduce radiation-induced DNA damage [28]. As such, additional *in vivo* studies will be required to assess if radiating tumours in the presence of lithium could increase risk of tumour recurrence from any surviving malignant cells. Cohen and co-workers observed an inverse trend of non-epithelial cancer incidence with lithium dose in patients treated with lithium carbonate for bipolar disorder [35]. Furthermore, Martinsson and co-workers found an increased risk of respiratory, gastrointestinal, and endocrine cancer in patients with bipolar disorder without lithium treatment [36]. Lithium has been used for treatment of bipolar or related disorders, also in children [37], and a recent study reported increased hippocampal volumes in lithium-treated adolescents [38]. Whereas lithium treatment for bipolar disorder is chronic, the herewith proposed treatment to prevent irradiation-induced late effects would be limited in time to the weeks of radiotherapy and could be easily monitored on a daily basis. The proposed treatment may be even be extended beyond the duration of radiotherapy to counteract degeneration and promote regeneration [9, 26, 30]. This has been tried in a handful of cases of TP53-mutated medulloblastoma [29], where lithium was used as a radiosensitizer, administered two weeks prior to, during and one month after radiotherapy, without any signs of toxicity [39] (Eric Bouffet, personal communication). Taken together, there is much evidence that lithium does not protect tumour cells, rather the opposite. With the information at hand we cannot distinguish between possible effects on quiescent and proliferating tumour cells though.

Irradiation has been shown to have multiple deleterious effects on the brain that are age- and radiation dose-dependent [40]. Neurocognitive impairments in both rodents and humans are associated with neural stem cell dysfunction, inflammation, and demyelination [41–43]. The neurogenic regions are particularly susceptible to irradiation [10, 32, 44]. Previously we showed that moderate, clinically relevant doses of irradiation to developing rodent brains caused extensive damage to neural stem and progenitor cells and significant reduction in the size of the neurogenic areas of the brain [32, 45]. Loss of neural stem and progenitor cells in the hippocampus is associated with impairment of cognitive function following irradiation in rodents, and possibly also in humans [41, 46, 47], and prevention of radiation-induced stem cell death can ameliorate cognitive dysfunction [6, 11, 15]. Two previous studies have demonstrated reduced neurogenesis and cognitive performance in irradiated mice and amelioration of these deficits by lithium [9, 23]. *In vitro*, lithium was shown to increase proliferation of hippocampal NSPCs and rescue irradiation-induced cell cycle arrest [28]. In this study, we found that lithium treatment prevented not only the radiation-induced cognitive deficits but also signs of hypothalamus–pituitary dysfunction without any apparent toxic effects. The doses used in this and other studies are in the same range as those used for humans, and no toxicity was observed. These results strongly suggest a potential of lithium treatment concomitant with cranial radiotherapy to ameliorate the irradiation-induced brain injury and cognitive deficits.

Inflammation plays an important role in brain injury, including after irradiation [45, 48, 49]. Radiation has been reported to activate microglia and to increase the expression of cytotoxic molecules such as pro-inflammatory cytokines and chemokines in the brain, and these have been implicated in radiotherapy-associated brain damage [9]. Neuroinflammation has been consistently implicated in the pathogenesis of radiation-induced cognitive decline [45, 48, 50, 51]. In the current study, lithium treatment prevented irradiation-induced acute inflammatory reactions 6 h after irradiation, and the same tendency was seen for cell death in the hippocampus. Because dying cells elicit inflammatory responses and because reduced cell death would result in attenuated inflammatory reactions, the decreased inflammation seen after lithium treatment could be secondary to reduced injury. This is supported by our finding that microglia activation *in vitro* by LPS was not affected by lithium treatment. Alternatively, it is possible that lithium-mediated inhibition of GSK3β in microglia indirectly restores inflammation-induced down-regulation of antioxidant capacity in astrocytes and thereby reduces cell death following oxidative stress [52].

In this study, lithium was administered before and after irradiation. Cell death was prevented or delayed at 6 hours and 24 hours after irradiation, and the inflammatory response, as indicated by cytokine and chemokine expression, was reduced at 6 hours after irradiation. These data indicate protective effects of lithium administration prior to irradiation. We previously showed that delayed lithium injection, 5 days after injury, could increase neurogenesis and decrease chronic inflammation in a model of hypoxic-ischemic brain injury [31]. So long as lithium administration during radiation does not promote subsequent tumour recurrence, our data suggest that cognitive benefits of lithium may be optimised if target serum concentrations are reached prior to radiotherapy, and maintained until a month or so after the end of the radiation treatment, as in the trial mentioned above, to maximise the benefit.

In addition to the direct effect of irradiation on neurogenesis and oxidative stress, cranial irradiation also has systemic effects on the entire body through radiation-induced endocrinopathy [53]. Growth hormone deficiency and hypothyroidism (central as well as peripheral) are common in survivors of paediatric brain tumours treated with radiotherapy [54, 55]. It has been shown in adult animals that cranial radiation induces deficits in pituitary weight and pituitary growth hormones, and that these changes are greater in younger adult rats [12]. The influence on the juvenile, still developing brain should, therefore, be even more pronounced. Our results show that the levels of pituitary-related hormones were substantially lower than in controls 5 weeks after irradiation, concurrent with reduced body weight gain in the irradiated rats. Lithium treatment prevented the TSH and GH reductions in the irradiated group, but not the reduced body weight gain. Increased BDNF levels, indicative of hypothalamic/pituitary stress [33], were also prevented by lithium treatment in irradiated rats. This indicates that lithium treatment can ameliorate irradiation-induced hypothalamus–pituitary functional deficits, and the underlying mechanisms need to be investigated further.

In summary, we found that treatment of rats with lithium in conjunction with cranial irradiation prevents NSPC death, stimulates NSPC proliferation and survival, reduces the cognitive decline and ameliorates the negative effects on the hypothalamus–pituitary axis. We also found that lithium is toxic to human paediatric brain tumour cells, but not neural stem cells, *in vitro*, and that lithium does not protect the brain tumour cells against irradiation. Lithium therefore holds great potential both as an adjuvant to radiotherapy as well as treatment to ameliorate the cognitive decline and possibly endocrine dysfunctions observed in survivors of paediatric malignant brain tumours.

## MATERIALS AND METHODS

### Tumour cell line culture

Human medulloblastoma wild-type TP53 cell lines (D283 MED, D425 MED, D458 MED, MED-MED8a) and TP53 mutant cell lines (DAOY and UW228-3) or primitive neuroectodermal tumour (PFSK-1) cell lines and a murine neural multipotent progenitor cell line (C17.2) were used. DAOY, D283MED and PFSK-1 were purchased from ATCC. The other cell lines were kindly provided by M Nistér (D425 MED, D458 MED, MED-MED-8a and UW228-3) or T Ringstedt (C17.2). The cell lines were cultured in minimum essential media (MEM; DAOY, D283 MED), RPMI-1640 (PFSK-1), Dulbecco's Modified Eagle's Medium (DMEM; MEB-MED-8A, C17.2), Richter's improved MEM with zinc/DMEM (IMEMZO/DMEM; D425 MED and D458 MED), DMEM/F12 (UW228-3), at 37°C in a humidified 5% CO_2_ atmosphere. Medium was supplemented with 15% (C17.2) or 10% foetal calf serum (FCS) (GIBCO), and 2 mM L-glutamine, 100 IU/mL penicillin G, and 100 μg/mL streptomycin. All media were purchases from GIBCO. Experiments were executed in Opti-MEM supplemented with glutamine and antibiotics. The identities of the human tumour cell lines were verified by short tandem repeat genetic profiling using the AmpFISTR^®^ Identifiler^®^ PCR Amplification Kit (Applied Biosystems) and all cell lines were used in passages below 25.

The effects of lithium chloride and radiation on cell growth were determined using a colorimetric formazan-based cell viability assay (WST-1; Roche) according to the manufactures description. Cells were seeded into 96-well plates (3,000 - 10,000 cells/well) left to attach and then treated with lithium chloride and incubated for 72 h; in the combined experiments, cells were first treated with lithium chloride (1 or 10 mM), irradiated using a ^60^Co radiation source (5 or 10 Gy) the day after and then incubated for another 72 h before analysis. All concentrations were tested in triplicate and the experiments were repeated at least three times. IC50 values were calculated as described earlier [56], using GraphPad Prism (GraphPad Software, San Diego, CA, USA) from log concentration - effect curves (cell viability %) by using nonlinear regression. Effect on cell viability was determined by using a colourimetric formazan-based cell viability assay (WST-1; Roche) after 72 hours incubation with lithium. WST-1 measures cell growth as well as inhibition, and is based on the cleavage of the tetrazolium salt WST-1 to formazan by cellular mitochondrial dehydrogenases. Expansion in the number of viable cells leads to an increase in the activity of the mitochondrial dehydrogenases, which in turn leads to increased amounts of formazan dye formed. The formazan dye produced by viable cells can be quantified by measuring the absorbance (at 440 nm). Background control absorbance was measured at 650 nM, absorbance at this wavelength was subtracted from the absorbance at 440 nm. Lithium was tested in concentrations ranging from 51.2 to 0.8 mM, diluted in seven twofold serial dilution steps. Cell viability was calculated absorbance in experimental wells in percent of control wells, with blank values subtracted.

### Primary microglia culture

Microglia cultures were prepared as previously described [57]. Briefly, brains were removed from 1- to 3-day-old C57Bl/6 pups, incubated for 15 min in 1 mg/ml trypsin, followed by 2 min of 1 mg/ml trypsin inhibitor, rinsed, dissociated and cultured in DMEM consisting of Hams F12 (50/50), supplemented with 10% FCS, 100 U/mL penicillin, 100 µg/mL streptomycin and 2 mM glutamine. The medium was replaced after 2 days. After 10-14 days, flasks were mechanically shaken for 60 min, 150 rpm to release microglia into the supernatant, subsequently subcultured into uncoated 96-well plates (15,000-17,000 cells/well). They were kept in 70% astrocyte-conditioned medium and 30% fresh DMEM/F12 supplemented with 10% FCS, 100 U/mL penicillin, 100 µg/mL streptomycin and 2 mM glutamine for 1-2 days to achieve microglia in a quiescent state. Medium was then changed to fresh medium as above or cells were incubated with different concentrations of lithium chloride (1-2 mM) for 24 h followed by 200 ng/ml lipopolysaccharide (LPS, Sigma). After another 24 h, medium was collected for TNF-α and IL-6 assay using the Duoset ELISA system according to the manufacturer's instructions (R&D, Minneapolis). Quantification of cell viability was performed using MTT reduction assay at 0.5 mg/mL for 1 h. The reaction was terminated by removing the media and freezing the plate at −80°C for at least 1 h. DMSO solvent was added to each well for 1h under shaking conditions at 37°C. The absorbance of each well was determined with an automated FLUOstar Optima reader at 570 nm with a reference filter at 630 nm.

### Animal irradiation procedure

A linear accelerator with 4 MV nominal photon energy and a dose rate of 2.3 Gy/min was used [58 ]. Postnatal day 11 (P11) male Wistar rat pups (Charles River, Germany) were anesthetized with an intraperitoneal injection of 50 mg/kg tribromoethanol. The animals were placed in a prone position with their head in the gantry of an expanded polystyrene bed. The whole brain was irradiated with a radiation field of 2 cm × 2 cm. A single absorbed dose of 6 Gy was administered. Using the linear-quadratic model[59] and an α/β ratio of 3 for late effects in normal brain tissue, the acute exposure of 6 Gy is approximately equivalent to 12 Gy when delivered in repeated 2 Gy fractions, thereby representing a clinically relevant, moderate radiation dose. The radiation dose administered to the brain and spinal cord of children with malignant brain tumours is usually higher. In the current medulloblastoma protocol (PNET 5), for example, a craniospinal dose of 23.4 Gy is combined with a 30.6 Gy boost to the tumour bed. From a practical point of view, for proof of principle, a single dose of 6-8 Gy is often used in rodent models [32]. Sham control animals were anaesthetized but not subjected to irradiation. This study was approved by the Gothenburg Committee of the Swedish Animal Welfare Agency (202-2012).

### Lithium administration

Lithium chloride (Aldrich, USA) was dissolved in normal saline and injected at a dose of 2 mmol/kg intraperitoneally at P7. For the short-term study, this was followed by 1 mmol/kg injections at 24 h intervals for four successive days until irradiation at P11. Pups were then sacrificed at 6 h or 24 h after irradiation. For the long-term study, the same initial protocol was used but after irradiation at P11 the rats were injected with 1 mmol/kg lithium chloride every 24 h for another 10 days [30]. These rats were sacrificed at 16 weeks after irradiation.

### BrdU administration

BrdU (5 mg/mL dissolved in 0.9% saline; Roche, Germany) was freshly prepared prior to use and injected intraperitoneally (50 mg/kg) once at P9 and once at P10. The rat pups were sacrificed 6 h or 24 h after irradiation at P11. For the long-term evaluation, BrdU injections were also given on P12 and P13 after irradiation at P11 and were sacrificed at 16 weeks after irradiation.

### Determination of cytokine and chemokine levels in the hippocampus

The hippocampus was collected from both control and irradiated pups at 6 h or 24 h after irradiation and was homogenized in 0.1 M PBS containing a cocktail of protease inhibitors (Roche) and 1% phosphatase inhibitor cocktail 2 (Sigma). The sample was homogenized and centrifuged at 10000 *g* for 15 min at 4°C. The supernatant was collected and stored at −80°C for the cytokine/chemokine assay. The total protein concentration was measured by using the BCA method. Concentrations of IL-1α, IL-1β, GRO/KC, and MCP-1 were simultaneously quantified using a MILLIPLEX^®^ MAP rat cytokine kit (Millipore).

### Pituitary-related hormone assay

Blood samples were collected from the tail in the morning around 10 am and placed at room temperature for about 60 min to coagulate and centrifuged at 1000 *g* for 10 *min*. The serum was then removed and stored at −80°C. The samples were analysed using the MILLIPLEX^®^ MAP rat pituitary magnetic bead panel assay kit (Millipore).

### Behavioural tests

Motor activity patterns were analysed with an open field system at about P28 and P60 of age [31]. Briefly, rats were placed into the centre of an unfamiliar 100 cm × 100 cm dark grey-coloured Plexiglas open-field arena, and the total movement distance was recorded for 20 min. The centre of the animal's body was defined as the point for tracking zone entries. The recorded video was analysed using the EthoVision 3.1 video-tracking software. The central zone was defined as a 30 cm × 30 cm area in the centre of the arena. The anxiety-related activity was assayed as the amount of time spent within the central zone.

Object recognition was tested the next day in the open-field box. The test consisted of two trials with an interval of 24 h. During the first trial, the animals were placed in the box containing two identical objects for 5 min. For the second trial, one of the objects presented in the first trial was replaced by a novel object. The animals were placed back in the box for 5 min, and the total time spent exploring each object was determined. Data are expressed as the recognition memory index [60].

### Immunohistochemistry

The paraffin embedded left hemisphere was cut into 5μm sagittal sections for haematoxylin and eosin (H&E) as well as cell death-related markers and stem/progenitor cell proliferation markers. Monoclonal rat anti-BrdU (1:200, AbD Serotec) or mouse anti-Ki67 (1:150, Novocastra) primary antibodies were applied and followed by biotinylated secondary antibody (1:500; Jackson ImmunoResearch) for 60 min at room temperature. Endogenous peroxidase activity was blocked with 3% H_2_O_2_ in PBS for 10 min. This was followed by incubation with Vectastain ABC. For the TUNEL labelling, the sections were incubated with 3% bovine serum albumin in 0.1 M Tris-HCl (pH 7.5) for 30 min followed by 50 µl TUNEL reaction mixture for 60 min at 37°C in a humidified chamber. After washing, the sections were mounted using Vectashield mounting medium.

For the long-term study, sagittal sections (30 μm) were used for stem cell proliferation and differentiation staining. BrdU labelling was performed on free-floating sections. After blocking with 3% donkey serum, the sections were incubated with rat anti-BrdU (1:200; AbD Serotec) overnight at 4°C. After rinsing, sections were incubated for 2 h at room temperature with biotinylated donkey anti-mouse (1:1000; Jackson ImmunoResearch). This was followed by incubation with Vectastain ABC and then detection solution. For phospho-histone H3 (PHH3) staining and brain lipid binding protein (BLBP) staining, the sections were incubated with rabbit anti-PHH3 (1:200, Upstate) and rabbit anti-BLBP (1:600, ABN14; Millipore) in blocking solution for 16 h at 4°C. After rinsing, sections were incubated for 2 h at room temperature with biotinylated donkey anti-rabbit secondary antibody (1:500).

The phenotypes of BrdU-labelled cells were determined using double immunofluorescent staining. BrdU staining was performed as above followed by either mouse anti-NeuN monoclonal antibody (1:200, Chemicon) and rabbit anti-S-100β (1:1000; Swant) in PBS. After washing, the sections were incubated with Alexa 488 donkey anti-rat IgG combined with Alexa 555 donkey anti-mouse IgG and Alexa 647 donkey anti-rabbit IgG (1:1000, Jackson ImmunoResearch) secondary antibodies. For double labelling of BrdU-GFAP, BrdU staining was performed as above and followed by goat anti-GFAP antibody (1:100; clone: SC-6170, Santa Cruz Biotechnology, Texas, USA) in PBS at 4°C for overnight. After washing, the sections were incubated with secondary antibodies, Alexa Fluor 488 donkey anti-rat IgG (H + L), combined with either Alexa Fluor 546 donkey anti-goat IgG (H + L) (1:1000, Jackson ImmunoResearch) at 20°C for 60 min. After washing, the sections were mounted using ProLong Gold antifade reagent with DAPI (ThermoFisher Scientific).

### Cell counting

The pyknotic cells as indicated by H&E staining and the TUNEL-positive cells were counted in the subgranular zone (SGZ) of the dentate gyrus (DG). The numbers of BrdU- or Ki67-labeled cells as well as total number of Iba1 positive and Iba1 positive with bushy morphology [31] in the short-term study or the numbers of BLBP-positive and PHH3-positive cells in the long-term study were counted in the SGZ. For the long-term study, BrdU-labelled cells were counted in the granular cell layer (GCL) using unbiased stereological counting techniques. For the immunofluorescent stainings, at least 50 BrdU-positive cells in the GCL were phenotyped using a confocal laser scanning microscope, and the ratio of BrdU-NeuN or BrdU-S100β double-labelled cells was calculated. The total number of newborn neurons or astrocytes in each sample was calculated based on the number of BrdU-positive cells and the ratio of double labelling.

### Creatinine measurement

Blood samples were collected from the tail or when the animals were sacrificed. Serum was isolated by centrifugation and stored at −80°C. The concentration of creatinine in the serum was detected with serum creatinine kit (Cat. KBO2-H1, Arbor Assays).

### GCL volume evaluation

The free-floating sections were stained with DAPI, and the area of the GCL in the DG was traced with a stereology system (Stereoinvestigator, MicroBrightField, Colchester, VT, USA). The volume was calculated by multiplying the sum of all areas by the section thickness (30 µm) and the serial factor (12).

### Statistics

All data were expressed as the mean ± SEM. Student's *t*-test was used when comparing two groups. A two-way ANOVA was used when comparing the effects of lithium treatment and irradiation, and this was followed by a post-hoc test. Repeated measures ANOVA was used when comparing body weight gain, pituitary hormones, and behavioural tests. The significance level was set at *p* < 0.05. IC_50_ values were calculated from log-concentration-effect curves in GraphPad Prism.
